# Asbestos-Related Pulmonary Spindle Cell Carcinoma Presenting With Ileus: An Autopsy Case Report

**DOI:** 10.7759/cureus.83634

**Published:** 2025-05-07

**Authors:** Kohei Yamane, Shiro Moriyama, Soichiro Ishikawa, Koji Fukutani, Akira Yamasaki

**Affiliations:** 1 Department of Respiratory and Infectious Diseases, Sanin Rosai Hospital, Yonago, JPN; 2 Department of Multidisciplinary Internal Medicine, School of Medicine, Faculty of Medicine, Tottori University, Yonago, JPN

**Keywords:** asbestos-related lung cancer, gastrointestinal metastasis, histopathological analysis, ileus, immunohistochemical findings, occupational asbestos exposure, postmortem diagnosis, pulmonary spindle cell carcinoma, rare lung cancer subtype, sarcomatoid carcinoma

## Abstract

We present the case of an 84-year-old male with a history of occupational asbestos exposure who initially developed ileus, which was later found to be related to an aggressive pulmonary malignancy. Imaging revealed extensive pleural thickening, peritoneal dissemination, and ileus. Postmortem examination confirmed a diagnosis of pulmonary spindle cell carcinoma (PSCC), supported by immunohistochemical analysis and the presence of numerous asbestos bodies in lung tissue. This case highlights the diagnostic complexity of rare thoracic malignancies, particularly when presenting with atypical features such as ileus. Further investigation is warranted to better understand asbestos-related carcinogenesis and to improve diagnostic and treatment approaches.

## Introduction

Pulmonary spindle cell carcinoma (PSCC) is defined by the WHO classification (5th edition) as a malignancy composed exclusively of spindle-shaped cells [1]. This tumor accounts for only 0.2-0.3% of all primary lung cancers and 13.3% of pulmonary sarcomatoid carcinomas [2,3]. The immunohistochemical staining shows positivity for the mesenchymal marker vimentin, along with confirmation of epithelial differentiation through markers such as cytokeratin and epithelial membrane antigen (EMA), which are considered useful for diagnosis [4]. PSCC is known for its rapid progression and poor prognosis [5]. Although asbestos exposure is a well-established risk factor for thoracic malignancies such as mesothelioma and lung cancer, its role in the pathogenesis of PSCC remains unclear. PSCC presenting initially with ileus is exceedingly uncommon, with only sporadic cases documented. Peritoneal spread from primary lung cancer is rare, and secondary ileus due to such dissemination is even rarer [6,7].

Here, we report a rare autopsy case of PSCC in a patient with documented asbestos exposure who presented with ileus as the initial symptom.

## Case presentation

An 84-year-old male presented to our hospital with lower abdominal pain. His medical history included hypertension, type 2 diabetes mellitus, and Alzheimer’s disease. Of note, he had significant occupational exposure to asbestos during his 20s to 40s, working in a boiler room on cargo ships. He was a non-smoker.

**Figure 1:**
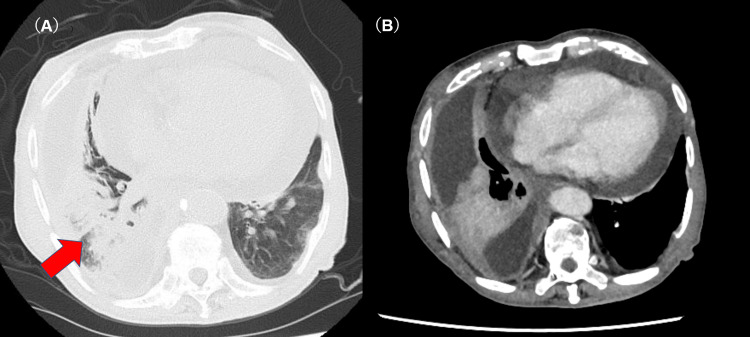
Chest CT images (A) An axial chest CT image shows an infiltrating shadow in the right lower lobe (arrow), right pleural effusion, and pleural thickening on the mediastinal side. (B) A contrast-enhanced axial CT image of the chest shows pericardial effusion.

The patient had been under observation for right pleural effusion for several years. Despite two thoracentesis procedures yielding bloody effusion, no malignant cells were detected. Imaging studies during his follow-up showed no significant progression until the onset of abdominal symptoms. Upon admission to our hospital, a computed tomography (CT) scan revealed a mass in the right lower lobe, right pleural effusion, pericardial effusion, and pleural thickening along the mediastinal side of the right upper and lower lobes, although no findings indicative of pleural plaques or interstitial lung disease were present (Figures 1A-1B).

**Figure 2:**
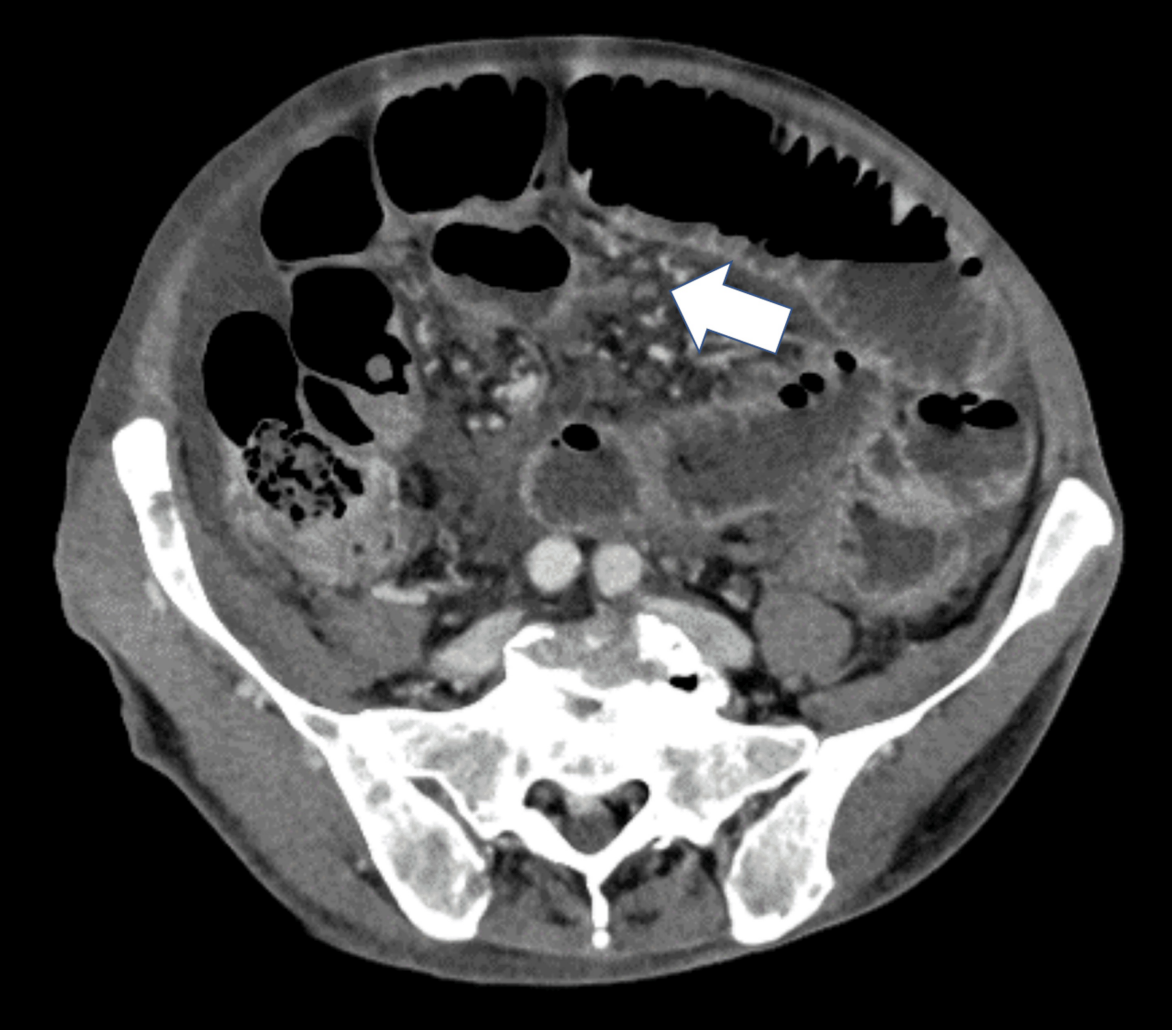
Abdominal CT Image A contrast-enhanced CT of the abdomen shows numerous peritoneal nodules (arrow) and findings indicative of ileus.

Abdominal imaging revealed multiple nodular lesions in the abdominal cavity, which were suggestive of peritoneal seeding, along with findings consistent with ileus (Figure 2).

While neither the patient nor his family reported a history of constipation, it is possible that symptoms were underreported due to the patient's cognitive impairment from advanced Alzheimer’s disease.

Pericardial drainage performed on the second day of hospitalization removed 560 mL of hemorrhagic effusion. Cytological analysis revealed malignant cells, although the histological subtype could not be determined. The patient's condition deteriorated rapidly, and he passed away on the eighth hospital day. Due to the rapid clinical deterioration, additional diagnostic procedures such as a biopsy of the lung lesion could not be performed. A pathological autopsy was performed with the family's consent.

**Figure 3:**
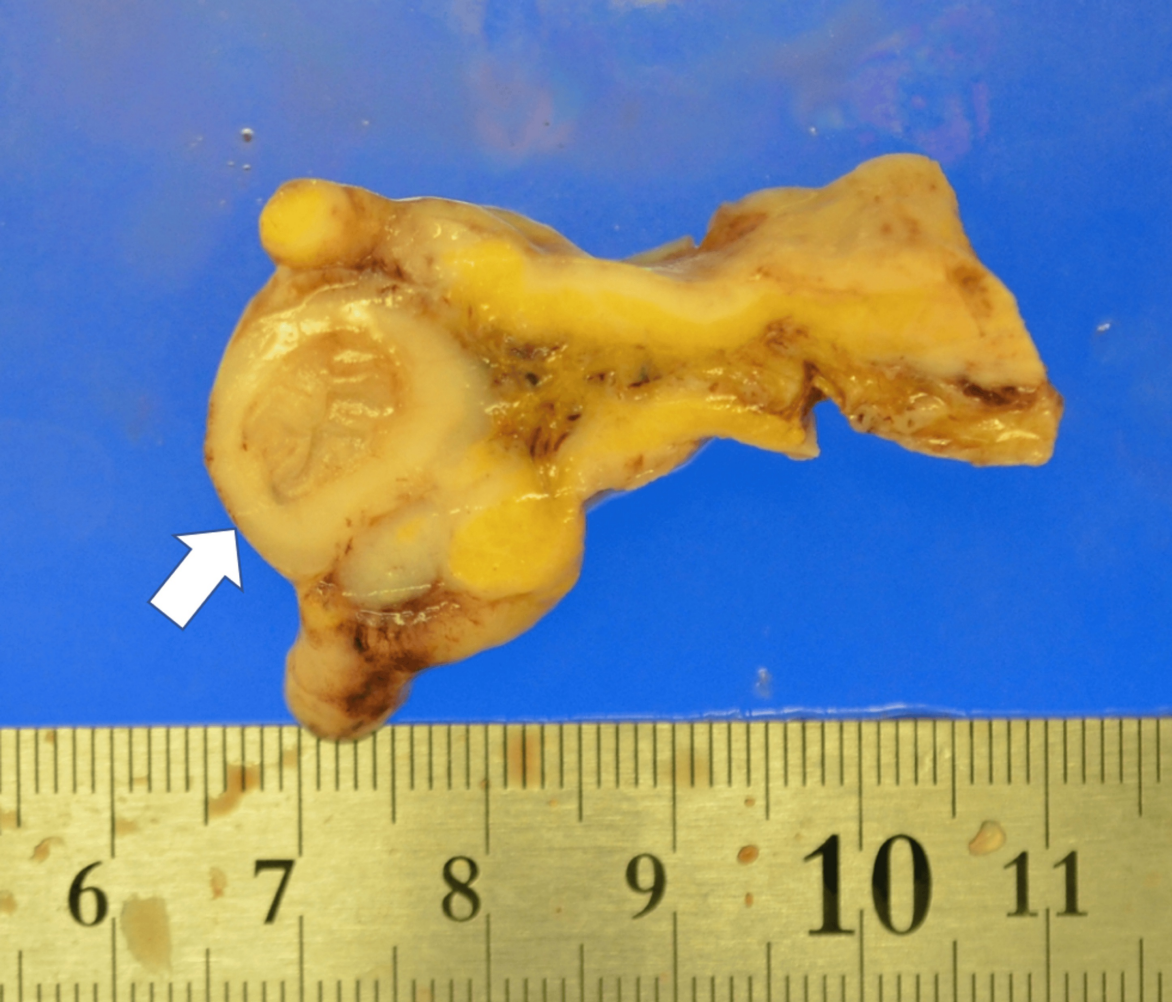
Sigmoid colon cross-section A cross-sectional image of the sigmoid colon from the autopsy specimen shows marked stenosis surrounded by metastatic lesions. The arrow indicates the site of luminal narrowing.

In the autopsy specimen, a relatively well-defined whitish tumor lesion was diffusely present along the mediastinal side of the lung, extending from the apex to the lower lobe. The heart predominantly exhibited white nodules on the posterior surface. Dissemination of white granular lesions was observed throughout the omentum, mesentery, and retroperitoneum. In the sigmoid colon, the disseminated lesions surrounded the area, causing marked stenosis, which was considered the cause of the ileus (Figure 3).

**Figure 4:**
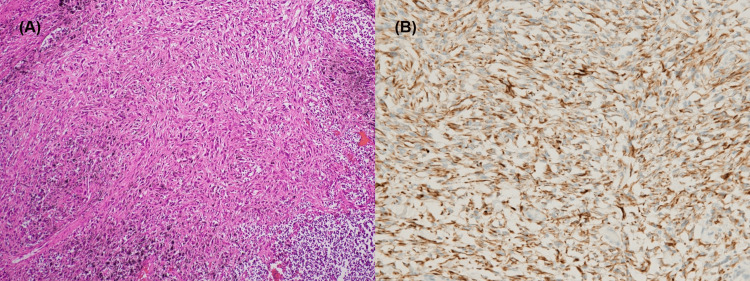
Representative histological and immunohistochemical images of the lung lesion (A) Hematoxylin and eosin staining of the lung lesion (200×) shows spindle-shaped atypical cells. (B) Immunohistochemical staining for CK7 (400×) demonstrates diffuse cytoplasmic positivity in the spindle cells from the same lesion.

Tumor cells in all affected organs exhibited a disorganized architecture composed predominantly of spindle-shaped atypical cells (Figures 4A-4B).

Immunohistochemical staining of the tumor tissue revealed positivity for the mesenchymal marker vimentin and epithelial markers including AE1/AE3, CK7, and EMA. In contrast, muscle-origin markers such as desmin and mesothelial markers such as calretinin, D2-40, WT-1, and CK5/6 were negative (Table 1).

**Table 1:** Summary of immunohistochemical staining results CK: cytokeratin; EMA: epithelial membrane antigen

Marker	Result
AE1/AE3	Positive
CK7	Positive
EMA	Positive
Vimentin	Positive
Calretinin	Negative
WT-1	Negative
D2-40	Negative
CK5/6	Negative
Desmin	Negative

Non-tumorous lung tissue revealed 12,183 asbestos bodies per gram of dried lung tissue, confirming asbestos-related PSCC. The cause of death was determined to be the progression of primary PSCC originating in the right lung, with extensive peritoneal dissemination and overall tumor progression.

## Discussion

PSCC is an aggressive variant of sarcomatoid carcinoma, characterized by spindle-shaped tumor cells. The median age of onset is 60 years, and it predominantly affects male smokers [4]. However, this case highlights an alternative etiological pathway involving significant asbestos exposure.

Asbestos exposure is a well-known risk factor for various lung cancer subtypes, particularly adenocarcinoma and squamous cell carcinoma [8,9]. However, its association with rare histological variants such as PSCC has been seldom reported. The Helsinki Criteria for asbestos-related diseases specifies thresholds for establishing causation, including an asbestos body count of ≥5,000 per gram of dried lung tissue [10]. In this case, the count of 12,183 asbestos bodies per gram far exceeded this threshold, supporting the diagnosis of asbestos-related lung cancer. The patient’s occupational history as a boiler room worker further reinforces this association.

Histopathological analysis revealed spindle-shaped atypical cells characteristic of PSCC. Immunohistochemically, the tumor was positive for AE1/AE3 and CK7, indicating epithelial differentiation. In contrast, mesothelial markers, including calretinin, WT-1, D2-40, and CK5/6, were all negative. Although the histopathologic distinction between PSCC and sarcomatoid malignant mesothelioma can be challenging, and sarcomatoid mesothelioma cannot be completely excluded, the uniform negativity of mesothelial markers argues against this possibility. According to the 2018 International Mesothelioma Interest Group (IMIG) guidelines, immunohistochemical panels containing both positive and negative mesothelial markers are essential for distinguishing malignant mesothelioma from other spindle cell malignancies [11]. Additionally, the tumor was centered in the lung parenchyma without forming a pleural-based mass, and only a superficial spread along the pleura was observed at autopsy. This distribution pattern is more consistent with a primary pulmonary tumor. Taken together with the markedly elevated asbestos body count, these findings strongly support the diagnosis of asbestos-related PSCC.

The clinical course of PSCC is typically aggressive, with rapid progression and poor survival rates [5]. Metastatic spread, including to the gastrointestinal tract, is not uncommon in sarcomatoid carcinomas. In this case, the patient developed extensive abdominal dissemination, leading to ileus. Gastrointestinal metastases are uncommon in primary lung cancers (8.8% of cases), but sarcomatoid carcinomas, including PSCC, have a higher metastatic tendency, with rates reaching up to 20% in related subtypes such as pleomorphic carcinoma [4,12].

The patient's rapid clinical deterioration underscores the diagnostic challenges of PSCC, as its aggressive behavior and atypical presentations frequently result in postmortem diagnoses despite advances in imaging and pathology. Early recognition and timely intervention are critical, yet remain difficult due to the rarity and heterogeneity of this histological subtype.

This case highlights the need for deeper investigation into the pathogenesis of asbestos-related sarcomatoid carcinomas. The molecular mechanisms by which asbestos fibers contribute to mutagenesis and sarcomatoid transformation in epithelial tumors like PSCC remain poorly understood. Given the extreme rarity of PSCC, collaborative multicenter research and large-scale case aggregation are indispensable for defining its clinicopathological features and for guiding future diagnostic and therapeutic strategies.

## Conclusions

In summary, this case underscores the clinical relevance of recognizing PSCC in patients presenting with unusual abdominal manifestations such as ileus. The significant asbestos burden observed suggests a potential etiologic role in the process of sarcomatoid transformation. To better understand and manage this highly uncommon and aggressive subtype, enhanced diagnostic vigilance and ongoing multidisciplinary collaboration are essential.
